# Gesundheitsinformationsverhalten und Gesundheitskompetenzen zur COVID-19-Schutzimpfung von Menschen in Deutschland – Befunde der CoSiD-Studie

**DOI:** 10.1007/s00103-022-03617-9

**Published:** 2022-11-03

**Authors:** Catherin Bosle, Boris Orth, Nadine Reibling, Christina Merkel, Carolin Muschalik, Ursula von Rüden

**Affiliations:** 1 Referat Q3 Evaluation, Methoden, Forschungsdaten, Bundeszentrale für gesundheitliche Aufklärung (BZgA), Maarweg 149–161, 50825 Köln, Deutschland; 2 Hochschule Fulda, Leipziger Straße 123, 36037 Fulda, Deutschland

**Keywords:** Informationsverhalten, Gesundheitskompetenz, COVID-19-Schutzimpfung, Impfabsicht, Repräsentativbefragung, Information behaviour, Health literacy, COVID-19 vaccination, Vaccination intention, Representative survey

## Abstract

**Hintergrund:**

Obwohl die COVID-19-Schutzimpfung schwere Krankheitsverläufe sowie Hospitalisierungen und Todesfälle reduziert, ist jede vierte bis fünfte Person in Deutschland nicht gegen COVID-19 geimpft. Um diese Menschen zu erreichen, werden effektive Informations- und Kommunikationsmaßnahmen benötigt. Dafür ist es wichtig, den subjektiven Informationsstand, das Informationsverhalten sowie die Gesundheitskompetenzen im Bereich der COVID-19-Schutzimpfung insbesondere von bisher Ungeimpften zu kennen.

**Methoden:**

Die dritte repräsentative Bevölkerungsbefragung (Nov./Dez. 2021; *n* = 4366) der CoSiD-Studie (Corona-Schutzimpfung in Deutschland) wurde als kombinierte Telefon- und Online-Befragung durchgeführt. Untersucht wurden bivariate Zusammenhänge zwischen Informationsstand, Informationsverhalten bzw. subjektiven Gesundheitskompetenzen und Impfstatus und -absicht. Zudem wurden multivariate Zusammenhänge soziodemografischer Merkmale mit subjektiven Gesundheitskompetenzen untersucht.

**Ergebnisse:**

Unentschlossene und eher Impfbereite berichten seltener einen guten subjektiven Informationsstand (46,1 %; 41,1 %) sowie die Kompetenz, Informationen zur COVID-19-Schutzimpfung zu bewerten (36,5 %; 38,8 %) und sich darauf basierend zu entscheiden (39,0 %; 35,9 %). Ungeimpfte ohne Impfabsicht schätzen Informationen häufiger als unglaubwürdig oder falsch ein (60,3 %). Menschen mit niedrigerem Bildungsniveau, Jüngere und Menschen mit Migrationshintergrund berichten geringere Gesundheitskompetenzen in Bezug auf die COVID-19-Schutzimpfung.

**Diskussion:**

Kommunikationsmaßnahmen zur Förderung der Gesundheitskompetenzen sollten gezielt Personen mit unsicherer Impfabsicht sowie Jüngere, Menschen mit niedrigerem Bildungsniveau und Menschen mit Migrationshintergrund zielgruppenspezifisch adressieren.

## Abstract (en)

**Background:**

Although COVID-19 vaccination reduces severe disease progression as well as hospitalisations and deaths, every fourth to fifth person in Germany is not vaccinated against COVID-19. Effective information and communication measures are needed to reach these people. For this, it is important to know the information behaviour as well as the health competences in the area of COVID-19 vaccination, especially of previously unvaccinated people.

**Methods:**

The third representative population survey (November/December 2021; *n* = 4366) of the CoSiD study (Corona vaccination in Germany) was conducted as a combined telephone and online survey. Bivariate correlations between reported information behaviour, subjective health literacy and vaccination status and intention are investigated. In addition, multivariate correlations of socio-demographic characteristics and subjective health literacy are analysed.

**Results:**

Undecided people and people that are more likely to vaccinate were overall less likely to report a good subjective level of information (46.1%; 41.1%) and competences in evaluating information about COVID-19 vaccination (36.5%; 38.8%) as well as decision making (39.0%; 35.9%). Unvaccinated people without vaccination intentions are more likely to report observing information rated as untrustworthy or wrong (60.3%). People with a lower level of education, younger people and people with a migration background report lower levels of heath literacy.

**Discussion:**

Communication measures to promote health literacy should specifically address people with uncertain vaccination intentions as well as younger people, people with lower levels of education and people with a migration background.

## Einleitung

Impfen ist eine der effektivsten Präventionsmaßnahmen gegen Infektionskrankheiten [1]. Durch die COVID-19-Schutzimpfung („corona virus disease 2019“) können Infektionen mit SARS-CoV‑2 („Severe acute respiratory syndrome coronavirus type 2“) bzw. COVID-19-Erkrankungen reduziert werden [2]. Außerdem werden schwere Krankheitsverläufe, Hospitalisierungen und Todesfälle bei geimpften Personen deutlich seltener beobachtet als bei ungeimpften Personen [3]. Dennoch machen nicht alle Bürgerinnen und Bürger von dem verfügbaren Impfangebot Gebrauch. Am 20.07.2022 waren laut digitalem Impfquotenmonitoring des Robert Koch-Instituts (RKI) mindestens 76,2 % der Bevölkerung durch die Impfung grundimmunisiert – wobei die Schätzung der Mindestimpfquote um bis zu 5 %-Punkte höher sein kann, da das hierfür herangezogene Meldesystem keine volle Erfassung der Impfungen bieten kann [4]. Um auch in Zukunft die Verbreitung von SARS-CoV‑2 weiter zu reduzieren, sodass Belastungen für Einzelne, die Gesellschaft und das Gesundheitssystem durch schwere oder tödliche Krankheitsverläufe, größere Ausbruchsgeschehen sowie damit einhergehende verhaltenspräventive Maßnahmen minimiert werden können, wird weiterhin eine möglichst hohe Impfquote empfohlen [5].

Eine große Herausforderung stellt hierbei die Erhöhung der Impfabsicht in der Bevölkerung dar. In einer Repräsentativbefragung im Rahmen der CoSiD-Studie (Corona-Schutzimpfung in Deutschland) im Juli 2021 zeigte sich die Gruppe der ungeimpften Personen in ihrer Impfabsicht heterogen. Während die Hälfte (51 %) der ungeimpften Befragten eine eher zögerliche Impfabsicht berichtet, wollten nur 18 % sich sicher impfen lassen und 31 % wollten sich sicher nicht impfen lassen [6]. Eine geringere Impfbereitschaft ist beispielsweise assoziiert mit einer Überschätzung der Impfnebenwirkungen oder einer Unterschätzung des Risikos einer COVID-19-Erkrankung, was darauf hindeutet, dass diese Personen zu wenig oder falsch informiert sind [7]. Deshalb bedarf es der (Weiter‑)Entwicklung von Informations- und Kommunikationsangeboten, welche das Vertrauen in Expertinnen und Experten fördern und dabei unterstützen, Vorteile und Risiken einer Impfung besser nachvollziehen zu können.

In Deutschland hat die Bundeszentrale für gesundheitliche Aufklärung (BZgA) die Aufgabe, die Bevölkerung über Infektionskrankheiten wie COVID-19 und Möglichkeiten zur Prävention zu informieren (siehe § 13 IfSGKoordinierungs-VwV^1^ und [8]). In diesem Kontext stellt sie allgemeinverständliche und zielgruppengerechte Informationen kostenfrei zur Verfügung (z. B. über Netzwerkarbeit, soziale Medien, Broschüren, Plakate, den BZgA-Shop www.shop.bzga.de, die Homepage www.infektionsschutz.de oder die Homepage www.longcovid-info.de). Dabei werden nicht nur die Allgemeinbevölkerung oder spezifische Teilgruppen, sondern auch Multiplikatorinnen und Multiplikatoren adressiert. Es ist das Ziel der BZgA, die Allgemeinbevölkerung durch ihre Angebote so zu unterstützen, dass diese in der Lage ist, individuell auf einer fundierten Wissensbasis Entscheidungen zum eigenen Schutzverhalten zu treffen. Hierfür werden auf der Basis der bestmöglichen qualitativen und quantitativen Evidenz und unter Einbezug von Expertinnen und Experten sowie von Multiplikatorinnen und Multiplikatoren bedarfsbezogene Informations- und Kommunikationsmaßnahmen entwickelt [9]. Dabei sollen insbesondere ungeimpfte Personen und Personen, deren Impfstatus ausläuft, adressiert werden, sodass die Impfabsicht und die Durchimpfungsraten in Deutschland erhöht werden können. Weitere wichtige Bausteine bilden das Informationsangebot „#ZusammenGegenCorona“ des Bundesministeriums für Gesundheit, das Programm „#ImpfenHilft“ der Bundesregierung mit den wichtigsten Gründen, warum sich Millionen von Menschen bereits für eine Impfung entschieden haben, sowie zielgruppenspezifische dialogorientierte Austauschformate, bei denen Stakeholder vor Ort und die Bevölkerung in direktem Kontakt sind.

Um die Impfabsicht besser verstehen zu können, ist es wichtig zu ermitteln, wie sich das Gesundheitsinformationsverhalten der Menschen in Bezug auf die COVID-19-Schutzimpfung gestaltet. Gesundheitsinformationsverhalten ist dadurch gekennzeichnet, welche Art (Inhalt und Vielfältigkeit) und Menge an Informationen gesucht, welche Methoden für die Suche eingesetzt und welche Quellen herangezogen werden [10]. Zu den Herausforderungen in der Pandemie gehört, dass die zur Verfügung stehenden Informationen einem kontinuierlichen und dynamischen wissenschaftlichen Erkenntnisgewinn unterliegen. Neben diesem dynamischen Prozess stellt auch die Verbreitung von falschen oder fehlerhaften Informationen, Gerüchten und Mythen eine große Herausforderung für die Entscheidungsfindung von Bürgerinnen und Bürgern dar. Durch soziale Medien werden impfkritische Meinungen kleinerer Gruppen effektiv verbreitet [11, 12]. Dies ist unter anderem auch deshalb zu berücksichtigen, weil die Wahrnehmung impfkritischer Informationen schnell die Risikowahrnehmung einer Impfung erhöhen kann [13, 14].

Die unterschiedlichen Prozesse der Informationsverbreitung und die große Menge an Informationen in der COVID-19-Pandemie waren sowohl für Laien als auch für Fachleute teilweise schwer einzuordnen und wurden entsprechend als „Infodemie“ bezeichnet [15]. Sie führten bei der Bevölkerung zu Unsicherheiten bezüglich der Inanspruchnahme einer Impfung. Entsprechend berichtete ein Großteil der Bevölkerung (55,2 %), durch die Menge an Informationen verunsichert zu sein [16], und jede fünfte Person fühlte sich noch im Sommer 2021 nur mäßig oder (sehr) schlecht über die COVID-19-Schutzimpfung informiert [6]. Gleichzeitig sank die Häufigkeit der Informationssuche, was mit einer allgemeinen „Pandemiemüdigkeit“ in Verbindung gestanden haben kann [17]. Dagegen war eine verstärkte Informationssuche mit einem besseren subjektiven Informationsstand verbunden, welcher wiederum positiv mit der Absicht, sich impfen zu lassen, assoziiert war [17]. Die Verbreitung von Falschinformationen war hingegen negativ mit der Impfabsicht assoziiert [18].

Um mit Gesundheitsinformationen umgehen zu können, bedarf es der Fähigkeit, die Informationen nicht nur zu finden und zu verstehen, sondern auch sie zu bewerten und darauf basierend Entscheidungen zu treffen. Diese Fähigkeiten werden zusammengefasst auch als „Gesundheitskompetenz“ beschrieben [19]. Die Bedeutung des Konzeptes der Gesundheitskompetenz erfährt in Deutschland mit dem „Nationalen Aktionsplan Gesundheitskompetenz“ zentrale Aufmerksamkeit [20]. Dessen Ziel ist es, mit geeigneten Angeboten die Gesundheitskompetenz der Menschen nachhaltig zu fördern und sie zu eigenen Entscheidungen in Bezug auf ihre Gesundheit zu befähigen. Unter anderem soll der Umgang mit Gesundheitsinformationen in den Medien erleichtert werden [20].

In Deutschland lag der Anteil von Personen mit geringer Gesundheitskompetenz im Jahr 2020 bei 58,8 % [21]. Insbesondere die Beurteilung von Gesundheitsinformationen und eine darauf basierende Entscheidungsfindung fällt vielen schwer [21]. Eine niedrige Gesundheitskompetenz steht häufig mit einem schlechteren Gesundheitsstatus [22], einer niedrigeren Lebensqualität [23] sowie weniger gesundheitsbewussten Verhaltensweisen wie etwa einer zu geringen physischen Aktivität [24] in Zusammenhang. Für den oben skizzierten komplexen Informationskontext in der COVID-19-Pandemie reicht es nicht aus, die allgemeine Gesundheitskompetenz zu betrachten [16]. Beispielsweise ist die coronaspezifische Gesundheitskompetenz im Verlauf der Pandemie zwar gestiegen, liegt aber bei circa einem Drittel der Bevölkerung (35,5 %) auf einem niedrigen Niveau [16]. Entsprechend sollen hier die spezifischen Gesundheitskompetenzen in Bezug auf die COVID-19-Schutzimpfung betrachtet werden.

Dieser Beitrag verfolgt insgesamt 3 Ziele. Das erste Ziel ist es, den Anteil der ungeimpften Personen in der Gesamtstichprobe und deren Impfabsicht zu beschreiben. Das zweite Ziel ist die Beschreibung der Art und Häufigkeit des berichteten Informationsverhaltens, des subjektiven Informationsstands sowie der subjektiven Gesundheitskompetenzen in Bezug auf die Corona-Schutzimpfung. Der Fokus liegt dabei auf der Verteilung dieser Faktoren nach Impfstatus und insbesondere nach der Impfintention ungeimpfter Personen. Als Drittes sollen Risikogruppen für niedrige Gesundheitskompetenzen in Bezug auf die COVID-19-Schutzimpfung ermittelt werden. Da durch den ablaufenden Impfstatus Auffrischungsimpfungen zunehmend relevanter wurden, wurden in diese Analysen auch geimpfte Personen eingeschlossen. Aus den Ergebnissen der Arbeit sollen zielgruppenspezifische Angebote abgeleitet werden.

## Methoden

### Studie, Datenerhebung und Stichprobe

Zur Beantwortung der Forschungsfragen wurden die Daten der dritten Erhebung der Begleitforschung zu den nationalen Kommunikationsmaßnahmen zur Corona-Schutzimpfung in Deutschland (CoSiD) ausgewertet. Die CoSiD-Studie ist eine deutschlandweite wiederholte Repräsentativbefragung der BZgA. Ziel ist es, das Wissen, die Einstellungen, die Informiertheit sowie das Verhalten der Allgemeinbevölkerung in Deutschland bezogen auf die COVID-19-Schutzimpfung repräsentativ abzubilden. Die dritte Befragung erfolgte vom 15.11. bis zum 08.12.2021. Weitere Informationen zur CoSiD-Studie sind auf der Website der BZgA veröffentlicht [6].

Für die Erhebung wurde das gesamte Gebiet der Bundesrepublik Deutschland eingeschlossen. Die Grundgesamtheit umfasste die deutschsprachige Wohnbevölkerung ab 16 Jahren in Privathaushalten. Die Datenerhebung erfolgte in Form eines Mixed-Mode-Ansatzes, welcher eine telefonische (CATI = Computer-Assisted Telephone Interviewing) und eine Online-Befragung (CAWI = Computer-Assisted Web Interviewing) kombinierte. Für die telefonische Befragung wurde eine repräsentative Haushaltsstichprobe auf Basis eines Dual-Frame-Ansatzes (Festnetz- und Mobilfunksample) zufällig gezogen. Die Stichprobe der Online-Befragung wurde zufällig aus einem aktiven Online-Access-Panel gezogen. Alle Befragten wurden vor Start des Interviews über die Ziele der Studie, die Freiwilligkeit der Teilnahme sowie die Anonymisierung und den Datenschutz aufgeklärt.

Der Fragebogen umfasste die Themen: persönlicher Gesundheitszustand, Risikowahrnehmung, Impfstatus und Impfabsicht, Impfeinstellung und Impfakzeptanz, wahrgenommene Normen, subjektiver Informationsstand und Informationsverhalten, Wahrnehmung von Impfkampagnen, Vertrauen in Institutionen und Politikverdrossenheit sowie Soziodemografie. Die telefonischen Interviews dauerten durchschnittlich 30 min, die Online-Befragungen dauerten durchschnittlich 24 min.

Insgesamt wurden in der dritten Erhebung *n* = 4366 Interviews durchgeführt. In jeder Erhebung der CoSiD-Studie wurde ein Basismodul erhoben, welches eine repräsentative Stichprobe von ca. 2000 Personen (hier *n* = 2058 Personen) aus der deutschsprachigen Wohnbevölkerung ab dem Alter von 16 Jahren in Privathaushalten abbildet. In der dritten Erhebung wurde als Zusatzmodul die Aufstockung um ungeimpfte Personen (*n* = 792) sowie um Personen mit Migrationshintergrund (*n* = 1516) realisiert.

### Untersuchte Variablen

#### Impfstatus und -absicht.

In der CoSiD-Studie wurden sowohl der Impfstatus („Haben Sie bereits Ihre Corona-Schutzimpfung erhalten?“) als auch die Impfabsicht („Haben Sie vor, sich gegen das Coronavirus impfen zu lassen?“) erhoben. Aus diesen beiden Variablen wurde ein kategorialer Indikator gebildet. Für diesen Indikator wurden alle Befragten mit mindestens einer Impfung zusammengefasst. Ungeimpfte Befragte wurden zusätzlich anhand der Impfabsicht in insgesamt 5 Kategorien unterteilt („auf jeden Fall impfen“, „eher impfen“, „unentschlossen“, „eher nicht impfen“, „auf keinen Fall impfen“).

#### Subjektiver Informationsstand und Informationsverhalten.

Der subjektive Informationsstand und das Informationsverhalten wurden über 3 eigens formulierte Indikatoren erhoben. Zum einem wurde der subjektive Informationsstand zur COVID-19-Schutzimpfung mit der Frage: „Was würden Sie sagen, wie gut fühlen Sie sich über die Corona-Schutzimpfung informiert?“, ermittelt. Die Antwortoptionen umfassten eine 5‑stufige Likert-Skala von „sehr schlecht“ bis „sehr gut“.

Die Häufigkeit der Informationssuche wurde durch die Frage: „Wie häufig haben Sie in den letzten 30 Tagen gezielt nach Informationen zur Corona-Schutzimpfung gesucht?“, erhoben. Die Antwortkategorien lagen auf einer 6‑stufigen Skala von „gar nicht“ bis „mehrmals täglich“.

Zudem wurde die subjektive Wahrnehmung von unglaubwürdigen oder falschen Informationen mit der Frage: „Wie häufig sind Ihnen in den vergangenen Wochen Informationen zur Sicherheit oder Wirksamkeit der Corona-Schutzimpfung begegnet, die Sie als unglaubwürdig oder falsch eingestuft haben?“, ermittelt. Antworten wurden auf einer 5‑stufigen Skala von „nie“ bis „sehr häufig“ erhoben.

#### Gesundheitskompetenzen im Bereich COVID-19-Schutzimpfung.

Die subjektiven Gesundheitskompetenzen im Bereich der COVID-19-Schutzimpfung wurden zudem durch die Befragten anhand von 4 Aussagen bewertet. Diese Aussagen umfassten: „Informationen über die Corona-Schutzimpfung zu finden, ist für mich einfach“, „Informationen über die Corona-Schutzimpfung zu verstehen, ist für mich einfach“, „Die Informationen über die Corona-Schutzimpfung zu beurteilen, ist für mich einfach“ sowie „Mich anhand der Informationen über die Corona-Schutzimpfung für oder gegen eine Impfung zu entscheiden, ist für mich einfach.“ Antworten wurden auf einer 5‑stufigen Likert-Skala von „stimme gar nicht zu“ bis „stimme voll und ganz zu“ erhoben. Diese 4 Aussagen orientieren sich an den 4 Kernkompetenzen des integrierten konzeptuellen Modells von Gesundheitskompetenz [19, 21].

#### Soziodemografische Indikatoren.

Als soziodemografische Variablen wurden aufgenommen: Alter (in Jahren), Geschlecht (männlich, weiblich, divers), Bildungsstand (maximal Hauptschulabschluss, Realschulabschluss, (Fach‑)Hochschulreife, (Fach‑)Hochschulabschluss), Haushaltsnettoeinkommen (keine Angabe, weniger als 60 %, 60 % bis weniger als 150 % sowie 150 % und mehr des Medians des Nettoäquivalenzeinkommens in Deutschland), Migrationshintergrund (Befragte oder mindestens ein Elternteil haben/hatten eine andere als die deutsche Staatsbürgerschaft) sowie Kinder unter 18 Jahren im Haushalt (ja, nein). Es wurde zudem die Region (ost- und westdeutsche Bundesländer) aufgenommen.

### Statistische Auswertung

In der vorliegenden Untersuchung wurden die Daten repräsentativ für die Allgemeinbevölkerung ausgewertet. Für die Gewichtung der Telefonstichprobe wurde eine Dual-Frame-Designgewichtung berechnet, die die unterschiedlichen Auswahlwahrscheinlichkeiten aufgrund unterschiedlich vieler Festnetz- und Mobilfunknummern sowie Personen im Haushalt ausgleicht. Zudem wurde die Gesamtstichprobe nach den Merkmalen Alter, Geschlecht, Schulbildung, Haushaltsgröße, Bundesland sowie konkretem Migrationshintergrund (gemäß den dafür jeweils aktuellsten Angaben des Statistischen Bundesamtes/Mikrozensus) gewichtet. Zuletzt wurde die Integration der Aufstockungen „Personen mit Migrationshintergrund“ und „Ungeimpfte“ durch Heruntergewichtung auf die jeweils realen Anteile berücksichtigt.

Das Modul „Complex Samples“ der Analysesoftware IBM SPSS Statistics 26.0. wurde für alle Auswertungen der Stichprobe genutzt. Uni- und bivariate Prävalenzen wurden für kategoriale Variablen in Prozentwerten abgebildet. Signifikante Gruppenunterschiede wurden anhand zweiseitiger 95 %-Konfidenzintervalle (KI) bestimmt. Zusammenhangsanalysen wurden mit multivariaten linearen Regressionsmodellen berechnet. Hierfür wurden die 4 Indikatoren zur Gesundheitskompetenz bezüglich der COVID-19-Schutzimpfung als abhängige Variablen und die soziodemografischen Indikatoren als Prädiktoren in das Modell eingefügt. In unseren Auswertungen werden die einzelnen Dimensionen der Gesundheitskompetenz getrennt betrachtet, weil ihnen unterschiedliche kognitive Prozesse zugrunde liegen, die auch mit der Qualität der Informationen zusammenhängen [19, 25]. Um Effekte des Erhebungsmodus zu berücksichtigen (z. B. durch Selektion in den Erhebungsmodus; siehe [26]), wurde für die Teilnahme an der Online- bzw. Telefonbefragung (CAWI vs. CATI) kontrolliert. Alle Analysen erfolgten auf Basis der gewichteten Stichprobe. Es werden die Regressionskoeffizienten inklusive Konfidenzintervalle berichtet. Das Signifikanzniveau wurde bei *p* < 0,05 angesetzt.

## Ergebnisse

Insgesamt wurden *n* = 4366 Personen in die Analysen aufgenommen. Die deskriptive Darstellung der Stichprobe ist in Tab. 1 zu finden. In der ungewichteten Stichprobe sind insbesondere ältere Menschen, Menschen mit niedrigerem Bildungsstand und Menschen ohne Migrationshintergrund unterrepräsentiert. Das wird durch die Gewichtung korrigiert. Etwa jede fünfte befragte Person (21,5 %) lebt in einem Haushalt mit einem Einkommen von weniger als 60 % des Medians des Nettoäquivalenzeinkommens, d. h. in einem Haushalt unterhalb der Armutsgrenze. Jede achte Person (12,6 %) verfügt über einen Migrationshintergrund und knapp jede vierte Person (24,2 %) lebt mit mindestens einem Kind unter 18 Jahren zusammen.

**Table 1:** 

		Absolute Häufigkeit (ungewichtetes *n*)	Absolute Häufigkeit (gewichtetes *n*)	Relative Häufigkeit (gewichtete %)
Alter	16 bis 25 Jahre	670	560	12,8
	26 bis 35 Jahre	804	646	14,8
	36 bis 45 Jahre	727	633	14,5
	46 bis 55 Jahre	767	758	17,4
	56 bis 65 Jahre	701	719	16,5
	66 bis 75 Jahre	441	507	11,6
	76 Jahre und älter	256	543	12,4
Geschlecht	Männlich	2111	2133	48,8
	Weiblich	2245	2227	51,0
	Divers	10	7	0,2
Bildung	Max. Hauptschulabschluss/Weiß nicht/Keine Angabe	649	837	19,2
	Realschulabschluss	1651	1929	44,2
	(Fach‑)Hochschulreife	907	719	16,5
	(Fach‑)Hochschulabschluss	1159	881	20,2
Nettoäquivalenzeinkommen	Keine Angabe	358	445	10,2
	< 60 % Median	1153	939	21,5
	60 % bis < 150 % Median	2330	2418	55,4
	≥ 150 % Median	525	564	12,9
Migrationshintergrund	Nein	2366	3817	87,4
	Ja	2000	549	12,6
Kinder unter 18 Jahren im Haushalt	Nein	3014	3308	75,8
	Ja	1352	1058	24,2
Region	Alte Bundesländer einschl. Westberlin	3603	3596	82,4
	Neue Bundesländer einschl. Ostberlin	763	770	17,6
Erhebungsmodus	CATI	1516	1948	44,6
	CAWI	2850	2418	55,4

*CATI* Computer-Assisted Telephone Interviewing, *CAWI* Computer-Assisted Web Interviewing

### Verteilung des Impfstatus und der Impfintention in der Gesamtstichprobe.

Insgesamt sind 12,3 % (95 %-KI: 11,9–12,8 %) der Stichprobe ungeimpft. Innerhalb dieser Gruppe der ungeimpften Personen wollen sich 8,1 % (95 %-KI: 6,4–10,2 %) auf jeden Fall impfen lassen. Der Anteil ungeimpfter Personen, die sich eher impfen lassen wollen, beträgt 8,3 % (95 %-KI: 6,8–10,2 %), während etwa jede fünfte ungeimpfte Person (21,4 % (95 %-KI: 18,9–24,1 %)) noch unentschlossen ist. Insgesamt geben 20,7 % (95 %-KI: 18,2–23,4 %) an, sich eher nicht impfen lassen zu wollen, und 41,5 % (95 %-KI: 38,3–44,8 %), dass sie sich auf gar keinen Fall impfen lassen möchten.

### Subjektiver Informationsstand, Informationsverhalten und subjektive Gesundheitskompetenzen in der Gesamtstichprobe sowie nach Impfstatus und -absicht.

**Figure 1:**
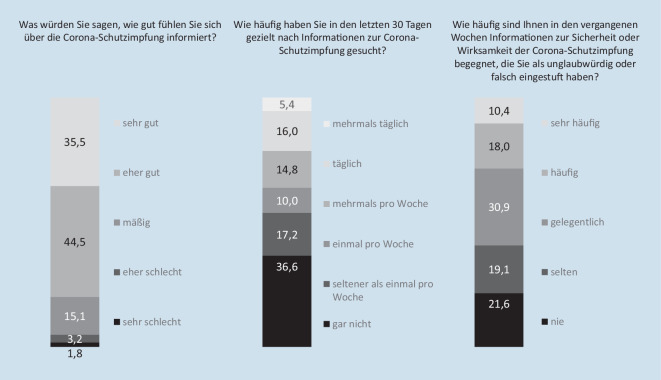

Die überwiegende Mehrheit der Befragten (80,0 %) fühlt sich sehr gut oder eher gut über die COVID-19-Schutzimpfung informiert und gut ein Drittel (36,6 %) gab an, in den letzten 30 Tagen nicht gezielt nach Informationen zur Impfung gesucht zu haben (Abb. 1). Ein Zehntel (10,4 %) hat in den vergangenen Wochen sehr häufig Informationen wahrgenommen, die er oder sie als unglaubwürdig oder falsch einstuft. Bei etwa einem Fünftel (21,6 %) war dies nie der Fall.

**Figure 2:**
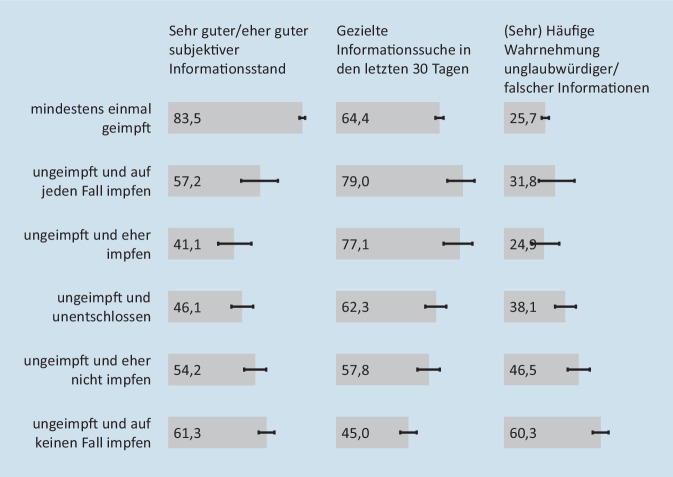

Insgesamt schätzen 83,5 % (95 %-KI: 81,6–85,3 %) der geimpften Personen und 61,3 % (95 %-KI: 56,3–66,1 %) derer, die sich auf keinen Fall impfen lassen wollen, ihren subjektiven Informationsstand zur COVID-19-Schutzimpfung als (sehr) gut ein (Abb. 2). Bei den eher Impfbereiten (41,1 % (95 %-KI: 31,1–51,8 %)) und den Unentschlossenen (46,1 % (95 %-KI: 39,3–52,9 %)) fühlen sich weniger als die Hälfte (sehr) gut informiert. Von denen, die sich auf jeden Fall bzw. eher impfen lassen wollen, berichten 79,0 % (95 %-KI: 69,3–86,2 %) bzw. 77,1 % (95 %-KI: 66,9–84,8 %) in den letzten 30 Tagen gezielt nach Informationen zur Schutzimpfung gesucht zu haben. Bei denen, die sich auf keinen Fall impfen lassen wollen, liegt der Anteil derer, die (sehr) häufig unglaubwürdige oder falsche Informationen zur Schutzimpfung wahrgenommen haben, mit rund 60,3 % (95 %-KI: 55,2–65,1 %) am höchsten.

**Figure 3:**
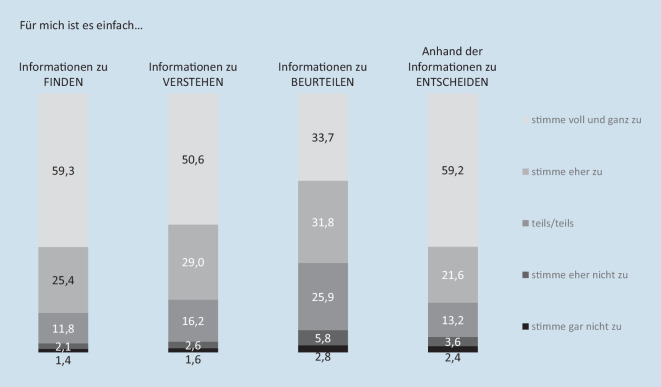

Die Mehrheit der Stichprobe schätzt die eigenen Gesundheitskompetenzen bezüglich der COVID-19-Schutzimpfung als gut bis sehr gut ein. Knapp 60 % stimmen voll und ganz zu, dass es für sie einfach sei, Informationen zu finden (59,3 %) und sich darauf basierend zu entscheiden (59,2 %; Abb. 3). 50,6 % stimmen voll und ganz zu, dass es für sie einfach sei, die Informationen zu verstehen. Der Aussage, dass die Informationen einfach zu beurteilen seien, stimmt hingegen nur ein Drittel (33,7 %) voll und ganz zu.

**Figure 4:**
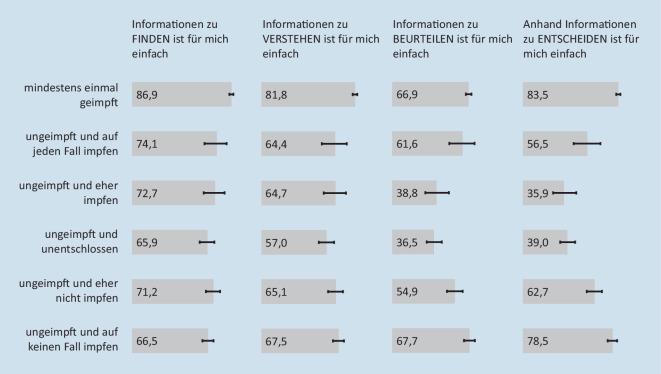

Gut 4/5 der Geimpften finden es einfach, Informationen zur Impfung zu finden (86,9 % (95 %-KI: 85,1–88,6 %)) und zu verstehen (81,8 % (95 %-KI: 79,7–83,8 %); Abb. 4). Den Ungeimpften fällt das – unabhängig von ihren Impfabsichten – signifikant seltener leicht (oberes 95 %-KI: je < 85,1 bzw. < 79,7). Insbesondere Unentschlossene und eher Impfbereite berichten seltener einen guten subjektiven Informationsstand (46,1 % (95 %-KI: 39,3–52,9 %); 41,1 % (95 %-KI: 31,1–51,8 %); Abb. 2) als auch die Gesundheitskompetenzen, Informationen zur COVID-19-Schutzimpfung zu beurteilen (36,5 % (95 %-KI: 30,2–43,3 %); 38,8 % (95 %-KI: 29,0–49,5 %)) und sich entsprechend zu entscheiden (39,0 % (95 %-KI: 32,6–45,7 %); 35,9 % (95 %-KI: 26,4–46,7 %); Abb. 4). Geimpften Menschen und denen, die sich auf keinen Fall impfen lassen wollen, fällt es am häufigsten leicht, die Informationen zu beurteilen und darauf basierend Entscheidungen zu treffen.

### Multivariate Zusammenhänge mit den subjektiven Gesundheitskompetenzen.

Tab. 2 zeigt die multivariaten Zusammenhänge zwischen den soziodemografischen Indikatoren und den Dimensionen der Gesundheitskompetenz. Die Fähigkeit, auf der Basis von Informationen Entscheidungen zu treffen, nimmt mit steigendem Alter zu (0,01; *p* < 0,000). Im Vergleich zu den Befragten mit (Fach‑)Hochschulabschluss fällt es denen, die maximal über einen Hauptschulabschluss verfügen, schwerer, Informationen zu finden (−0,15; *p* = 0,027) und zu verstehen (−0,16; *p* = 0,030), während Ersteres Personen mit (Fach‑)Hochschulreife leichter fällt (0,11; *p* = 0,038). Menschen mit Migrationshintergrund fällt es weniger leicht, Informationen zu finden (−0,12; *p* = 0,001), zu verstehen (−0,17; *p* = 0,017) und anhand der Informationen zu einer Entscheidung zu kommen (−0,03; *p* < 0,000), als Menschen ohne Migrationshintergrund. Dagegen können keine signifikanten Zusammenhänge zwischen den Gesundheitskompetenzen und dem Geschlecht oder dem Einkommen festgestellt werden.

**Table 2:** 

Es ist einfach für mich, …	Informationen zu FINDEN			Informationen zu VERSTEHEN			Informationen zu BEURTEILEN			Anhand Infos zu ENTSCHEIDEN		
	%	β (95 %-KI)	*p*	%	β (95 %-KI)	*p*	%	β (95 %-KI)	*p*	%	β (95 %-KI)	*p*
*Konstante*	–	4,12 (3,93; 4,32)	0,000	–	4,13 (3,92; 4,35)	0,000	–	3,64 (3,39; 3,88)	0,000	–	3,85 (3,64; 4,06)	0,000
*Alter*	–	0,00 (0,00; 0,01)	0,004	–	0,00 (0,00; 0,00)	0,182	–	0,00 (0,00; 0,01)	0,006	–	0,01 (0,00; 0,01)	0,000
*Geschlecht*												
Männlich	85,1	0,04 (−0,03; 0,11)	0,297	80,3	−0,02 (−0,10; 0,07)	0,706	67,8	0,07 (−0,02; 0,17)	0,114	80,9	0,03 (−0,05; 0,11)	0,465
Weiblich^a^	84,3	–	–	79,0	–	–	63,2	–	–	80,6	–	–
*Bildung*												
Max. Hauptschulabschluss/WN/KA	79,3	−0,15 (−0,27; −0,02)	0,027	75,0	−0,16 (−0,30; −0,01)	0,030	63,0	−0,02 (−0,17; 0,14)	0,831	79,0	0,01 (−0,12; 0,14)	0,879
Realschulabschluss	84,3	−0,03 (−0,13; 0,06)	0,498	78,6	−0,08 (−0,19; 0,04)	0,179	65,0	0,01 (−0,11; 0,13)	0,884	80,8	0,05 (−0,06; 0,16)	0,338
(Fach‑)Hochschulreife	88,3	0,11 (0,01; 0,21)	0,038	81,4	−0,03 (−0,14; 0,09)	0,636	67,3	0,12 (−0,02; 0,25)	0,094	81,5	0,13 (0,00; 0,26)	0,052
(Fach‑)Hochschulabschluss^a^	87,6	–	–	84,9	–	–	67,4	–	–	81,7	–	–
*Nettoäquivalenzeinkommen*												
Keine Angabe	86,8	−0,09 (−0,24; 0,07)	0,264	79,4	−0,07 (−0,26; 0,12)	0,463	66,4	0,01 (−0,19; 0,21)	0,907	87,7	0,05 (−0,11; 0,21)	0,515
< 60 % Median	79,3	−0,20 (−0,33; −0,07)	0,002	75,0	−0,12 (−0,27; 0,03)	0,112	60,6	−0,14 (−0,30; 0,02)	0,091	75,9	−0,13 (−0,27; 0,02)	0,089
60 % bis < 150 % Median	85,3	−0,09 (−0,20; 0,02)	0,091	80,6	−0,06 (−0,19; 0,07)	0,363	65,8	−0,06 (−0,20; 0,08)	0,381	80,7	−0,07 (−0,19; 0,05)	0,240
≥ 150 % Median^a^	89,5	–	–	83,4	–	–	71,3	–	–	83,7	–	–
*Migrationshintergrund*												
Ja	80,0	−0,12 (−0,19; −0,05)	0,001	76,8	−0,09 (−0,16; −0,02)	0,017	61,4	−0,05 (−0,14; 0,03)	0,201	67,7	−0,3 (−0,38; −0,22)	0,000
Nein^a^	85,4	–	–	80,0	–	–	66,1	–	–	82,6	–	–
*Kinder unter 18 Jahren im Haushalt*												
Ja	83,3	0,04 (−0,05; 0,13)	0,419	76,5	−0,07 (−0,17; 0,02)	0,144	63,2	−0,01 (−0,12; 0,10)	0,873	76,0	−0,03 (−0,14; 0,07)	0,571
Nein^a^	85,1	–	–	80,6	0,00 (0,00; 0,00)	0,182	66,2	–	–	82,3	–	–
*Region*												
Alte Bundesländer einschl. Westberlin	85,9	0,15 (0,05; 0,26)	0,005	80,7	−0,02 (−0,10; 0,07)	0,706	66,2	0,07 (−0,06; 0,19)	0,303	81,0	0,06 (−0,05; 0,17)	0,308
Neue Bundesländer einschl. Ostberlin^a^	78,9	–	–	74,8	–	–	62,3	–	–	79,5	–	–
*Befragungsmethode*												
CATI	86,8	0,19 (0,11; 0,27)	0,000	80,9	−0,16 (−0,30; −0,01)	0,030	64,9	−0,04 (−0,14; 0,07)	0,481	85,0	0,22 (0,13; 0,30)	0,000
CAWI^a^	83,0	–	–	78,7	–	–	66,0	–	–	77,4	–	–

Die Prozentangaben beziehen sich auf den jeweiligen Anteil der Befragten, der voll und ganz bzw. eher zustimmte

*β (95* *%-KI)* Regressionskoeffizient mit 95 %-Konfidenzintervall, *CATI* Computer-Assisted Telephone Interviewing, *CAWI* Computer-Assisted Web Interviewing, *KA* keine Angabe, *WN* weiß nicht

^a^Referenzgruppe im Regressionsmodell

## Diskussion

Die Ergebnisse der dritten Erhebung der CoSiD-Studie Ende 2021 zeigen eine geringere Impfabsicht in der Gruppe der ungeimpften Personen als in früheren Erhebungen [6]. Während der Anteil der Personen mit Unsicherheiten relativ konstant geblieben ist (von 51 % zu 50 %), berichtet ein deutlich kleinerer Anteil Ungeimpfter, dass sie sich auf jeden Fall noch impfen lassen wollen (von 18 % zu 8 %), und anteilig mehr Ungeimpfte wollen sich auf gar keinen Fall impfen lassen (von 31 % zu 42 %). Eine geringere Impfabsicht könnte darin begründet liegen, dass sich seit der letzten Befragung insbesondere Personen mit einer positiven Einstellung bereits haben impfen lassen und dadurch der Anteil der impfkritischen Personen größer geworden ist. Eine vergleichbare Repräsentativbefragung, die noch früher (im Spätsommer 2020) stattfand und auch eine große Stichprobe von Personen umfasste, die ein Jahr später bereits geimpft waren, ermittelte ebenfalls eine deutlich höhere Impfabsicht im Vergleich zu den aktuellsten CoSiD-Daten [16].

Unsere Ergebnisse zeigen zudem, dass sich der subjektive Informationsstand, das Informationsverhalten und die subjektiven Gesundheitskompetenzen in Bezug auf die COVID-19-Schutzimpfung je nach Impfstatus und -absicht unterscheiden. Insbesondere Unentschlossene und eher Impfbereite fühlen sich seltener gut informiert und berichten ebenfalls seltener gute Kompetenzen bezüglich der Informationsbeurteilung und der Entscheidungsfindung zur COVID-19-Schutzimpfung. Es ist daher weiterhin wichtig, für impfbereite und unentschlossene Personen, verlässliche und qualitätsgesicherte Informationsangebote vorzuhalten, die die Informationsbeurteilung und Entscheidungskompetenz fördern. Da die Informationsbeurteilung auch unabhängig von Impfstatus und -absicht deutlich schwerer fällt als das Finden und Verstehen von Informationen (siehe auch [16, 27]), besteht neben Informations- und Kommunikationsmaßnahmen ein Bedarf an Angeboten zur Entscheidungsfindung (wie z. B. onlinebasierte Entscheidungshilfen, Beratungsgespräche, Stärkung einer multiplikatorenbasierten Ansprache).

Menschen, die sich auf keinen Fall impfen lassen wollen, fühlen sich ähnlich gut informiert wie bereits geimpfte Menschen und schätzen auch ihre Gesundheitskompetenzen in Bezug auf die COVID-19-Schutzimpfung ähnlich hoch ein. Eine Erhebung, die vor der Impfstofffreigabe im Jahr 2020 durchgeführt wurde, zeigte dagegen auf, dass eine geringere Impfabsicht mit einer geringeren coronaspezifischen Gesundheitskompetenz einhergeht [16]. Außerdem nehmen Personen, die sich nicht impfen lassen wollen, am häufigsten unglaubwürdige oder Falschinformationen wahr. Die vorliegenden Analysen können nicht beantworten, welche Informationen als unglaubwürdig oder falsch wahrgenommen werden und ob diese auch faktisch falsch sind. Jedoch ist die Verbreitung tatsächlicher Falschinformationen negativ mit der Impfabsicht assoziiert [18]. Hieran zeigt sich, wie wichtig das Vertrauen der Bevölkerung in die Informationen von Gesundheitsinstitutionen und von Expertinnen und Experten ist. Dialogorientierte Informationsangebote und eine verstärkte Richtigstellung von Falschinformationen sind diesbezüglich essentiell.

Befragte mit geringerer Impfabsicht suchen seltener gezielt nach Informationen zur COVID-19-Schutzimpfung. Es ist somit wichtig, zu ermitteln, welche Informationskanäle diese Menschen nutzen, um sie gezielt ansprechen zu können. Ärztinnen und Ärzte gehören zu den häufigsten und vertrauenswürdigsten Quellen für Gesundheitsinformationen [28]. Daneben sind auch Massenmedien und lokale Zeitschriften häufig genutzte Quellen, welche, insofern ihnen vertraut wird, die Impfabsicht steigern können [29]. Dagegen können Nutzung und Vertrauen in Medien, welche vermehrt Falschinformationen streuen, einen negativen Einfluss haben [29]. Entsprechend müssen zielgruppenspezifische Angebote zur Medienkompetenz (weiter-)entwickelt werden, damit Güte und Verlässlichkeit insbesondere von Online-Informationen und deren Quellen besser bewertet werden können.

Zuletzt zeigen unsere Ergebnisse, dass insbesondere jüngere Menschen, Menschen mit niedrigerem Bildungsniveau sowie Menschen mit Migrationshintergrund geringere Gesundheitskompetenzen bezüglich der COVID-19-Schutzimpfung berichten, während die Merkmale Geschlecht und Einkommen kaum eine oder keine Rolle spielen. Für diese Gruppen sollten Kommunikationsmaßnahmen entwickelt und evaluiert werden, sodass sichergestellt wird, dass diese Gruppen erreicht und die Informationen verstanden werden.

Die präsentierten Ergebnisse müssen unter Einbezug verschiedener Eigenschaften der CoSiD-Daten interpretiert werden. Die CoSiD-Studie ermöglicht aufgrund des Studiendesigns (z. B. bundesweite Befragung, Mixed-Mode-Ansatz, Gewichtung) bevölkerungsrepräsentative Aussagen über die Impfabsicht und weitere Indikatoren. Eine Einschränkung ist allerdings, dass die Daten nur querschnittlich erhoben wurden und somit keine kausalen Schlussfolgerungen zulassen. Die Daten basieren zudem auf subjektiven Einschätzungen, wodurch die Ergebnisse verzerrt sein können. Beispielsweise ist zu vermuten, dass durch die Erhebung der subjektiven Gesundheitskompetenzen eine Überschätzung vorliegt [30].

## Fazit

Impfstatus und -absicht stehen in Zusammenhang mit dem subjektiven Informationsstand, dem Informationsverhalten und den subjektiven Gesundheitskompetenzen hinsichtlich der COVID-19-Schutzimpfung. Die (Weiter‑)Entwicklung von Kommunikationsmaßnahmen zur Förderung der Gesundheitskompetenzen in Bezug auf die COVID-19-Schutzimpfung sowie die Richtigstellung von Falschinformationen sind weiterhin große Herausforderungen im Hinblick auf die Erhöhung der Impfquote. Zur Entwicklung geeigneter Maßnahmen sollten gezielt Zugänge und Barrieren von Personen mit unsicherer Impfabsicht identifiziert werden. Dabei sollten insbesondere auch Jüngere, Menschen mit niedrigerem Bildungsniveau sowie Menschen mit Migrationshintergrund zielgruppenspezifisch adressiert werden (z. B. durch Einsatz dialogischer Methoden durch Multiplikatorinnen und Multiplikatoren).

## Footnotes

1 Allgemeine Verwaltungsvorschrift über die Koordinierung des Infektionsschutzes in epidemisch bedeutsamen Fällen (Verwaltungsvorschrift-IfSG-Koordinierung – IfSGKoordinierungs-VwV), § 13 Absatz 1–5.

## References

[1] Robert Koch Institut (2022) Impfen. https://www.rki.de/DE/Content/Infekt/Impfen/impfen_node.html. Zugegriffen: 22. Juli 2022

[2] Bundesministerium für Gesundheit (2022) Zusammen gegen Corona – Wirksamkeit, Risiken und Nebenwirkungen. https://www.zusammengegencorona.de/faqs/impfen/risiken-und-nebenwirkungen/. Zugegriffen: 22. Juli 2022

[3] Lahne H, Grahl A, Streibl B (2022). COVID-19-Impfung senkt das Risiko für Infektion, schwere Krankheitsverläufe und Tod. Epid Bull.

[4] Robert Koch Institut (2022) Impfdashboard – Aktueller Impfstatus. https://impfdashboard.de/. Zugegriffen: 22. Juli 2022

[5] Robert Koch Institut (2022) Infektionsschutz – Allgemeines (Stand: 20.07.2022). https://www.rki.de/SharedDocs/FAQ/COVID-Impfen/FAQ_Liste_Allgemeines.html. Zugegriffen: 20. Juli 2022

[6] Bundeszentrale für gesundheitliche Aufklärung (2021). Begleitforschung zur Kommunikation der Corona-Schutzimpfung in Deutschland (CoSiD) – Ergebnisse einer Repräsentativbefragung der Allgemeinbevölkerung im Juli 2021. BZgA-Forschungsbericht.

[7] Haug S, Schnell R, Scharf A, Altenbuchner A, Weber K (2021). Bereitschaft zur Impfung mit einem COVID-19-Vakzin – Risikoeinschätzung, Impferfahrungen und Einstellung zu Behandlungsverfahren. Präv Gesundheitsf.

[8] Robert Koch Institut (2020) Vorbereitungen auf Maßnahmen in Deutschland Version 1.0 (Stand 04.03.2020). Ergänzung zum Nationalen Pandemieplan – COVID-19 – neuartige Coronaviruserkrankung. https://www.rki.de/DE/Content/InfAZ/N/Neuartiges_Coronavirus/Ergaenzung_Pandemieplan_Covid.pdf?__blob=publicationFile. Zugegriffen: 22. Juli 2022

[9] Von Rüden U, Spura A, Horstmann S (2021). Bedarfsbezogene Kommunikationsstrategie der Bundeszentrale für gesundheitliche Aufklärung (BZgA) während der COVID-19-Pandemie. Bundesgesundheitsblatt Gesundheitsforschung Gesundheitsschutz.

[10] Lambert SD, Loiselle CG (2007). Health information—seeking behavior. Qual Health Res.

[11] Kata A (2012). Anti-vaccine activists, Web 2.0, and the postmodern paradigm—An overview of tactics and tropes used online by the anti-vaccination movement. Vaccine.

[12] Meyer C, Reiter S (2004). Impfgegner und Impfskeptiker. Bundesgesundheitsblatt Gesundheitsforschung Gesundheitsschutz.

[13] Betsch C, Renkewitz F, Betsch T, Ulshöfer C (2010). The influence of vaccine-critical websites on perceiving vaccination risks. J Health Psychol.

[14] Nan X, Madden K (2012). HPV vaccine information in the blogosphere: How positive and negative blogs influence vaccine-related risk perceptions, attitudes, and behavioral intentions. Health Commun.

[15] Germani F, Biller-Andorno N (2021). The anti-vaccination Infodemic on social media: a behavioral analysis. PLoS One.

[16] Okan O, Bollweg TM, Bauer U, Hurrelmann K, Janner C, Schaeffer D (2021). Trendstudie zur coronaspezifischen Gesundheitskompetenz: Ergebnisse der zweiten Erhebung der HLS-COVID-19 Studie.

[17] Schöberl S, Kieweg P (2021). Veränderungen im Informationsverhalten in der Corona-Krise und ihre Auswirkungen auf die Sichtweisen junger Menschen. kommges.

[18] Pierri F, Perry BL, Deverna MR (2022). Online misinformation is linked to early COVID-19 vaccination hesitancy and refusal. Sci Rep.

[19] Sørensen K, Van Den Broucke S, Fullam J (2012). Health literacy and public health: A systematic review and integration of definitions and models. BMC Public Health.

[20] Schaeffer D, Hurrelmann K, Bauer U, Kolpatzik K (2020). Nationaler Aktionsplan Gesundheitskompetenz. Die Gesundheitskompetenz in Deutschland stärken.

[21] Schaeffer D, Berens E-M, Gille S (2021). Gesundheitskompetenz der Bevölkerung in Deutschland vor und während der Corona Pandemie: Ergebnisse des HLS-GER 2.

[22] Berkman ND, Sheridan SL, Donahue KE, Halpern DJ, Crotty K (2011). Low health literacy and health outcomes: an updated systematic review. Ann Intern Med.

[23] Zheng M, Jin H, Shi N (2018). The relationship between health literacy and quality of life: a systematic review and meta-analysis. Health Qual Life Outcomes.

[24] Buja A, Rabensteiner A, Sperotto M (2020). Health literacy and physical activity: a systematic review. J Phys Act Health.

[25] Magasi S, Durkin E, Wolf MS, Deutsch A (2009). Rehabilitation consumers’ use and understanding of quality information: a health literacy perspective. Arch Phys Med Rehabil.

[26] Bowyer BT, Rogowski JC (2017). Mode matters: Evaluating response comparability in a mixed-mode survey. Polit Sci Res Methods.

[27] Dadaczynski K, Okan O, Messer M (2021). Digital health literacy and web-based information-seeking behaviors of university students in Germany during the COVID-19 pandemic: cross-sectional survey study. J Med Internet Res.

[28] Baumann E, Czerwinski F, Rosset M, Seelig M, Suhr R (2020). Wie informieren sich die Menschen in Deutschland zum Thema Gesundheit? Erkenntnisse aus der ersten Welle von HINTS Germany. Bundesgesundheitsblatt Gesundheitsforschung Gesundheitsschutz.

[29] Gehrau V, Fujarski S, Lorenz H, Schieb C, Blöbaum B (2021). The impact of health information exposure and source credibility on COVID-19 vaccination intention in germany. Int J Environ Res Public Health.

[30] Domanska OM, Firnges C, Bollweg TM, Sørensen K, Holmberg C, Jordan S (2018). Do adolescents understand the items of the European Health Literacy Survey Questionnaire (HLS-EU-Q47)–German version? Findings from cognitive interviews of the project “Measurement of Health Literacy Among Adolescents” (MOHLAA) in Germany. Arch Public Health.

